# Optimizing chitosan derived from *Metapenaeus affinis*: a novel anti-biofilm agent against *Pseudomonas aeruginosa*

**DOI:** 10.1186/s13568-024-01732-1

**Published:** 2024-06-29

**Authors:** Anali Riahi, Hadideh Mabudi, Elahe Tajbakhsh, Laleh Roomiani, Hasan Momtaz

**Affiliations:** 1 Department of Microbiology, Faculty of Basic Sciences, Shahrekord Branch, Islamic Azad University, Shahrekord, Iran; 2 Department of Fisheries, Ahvaz Branch, Islamic Azad University, Ahvaz, Iran

**Keywords:** Virulence factors, Chitosan, Antibacterial activity, Biofilm, Quorum sensing

## Abstract

*Pseudomonas aeruginosa* is a commonly found Gram-negative bacterium in healthcare facilities and is renowned for its ability to form biofilms and its virulence factors that are controlled by quorum sensing (QS) systems. The increasing prevalence of multidrug-resistant strains of this bacterium poses a significant challenge in the field of medicine. Consequently, the exploration of novel antimicrobial agents has become a top priority. This research aims to optimize chitosan derived from white shrimp (*Metapenaeus affinis*) using the Response Surface Methodology (RSM) computational approach. The objective is to investigate chitosan’s potential as a solution for inhibiting QS activity and biofilm formation in *P. aeruginosa* ATCC 10,145. Under optimized conditions, chitin was treated with NaOH (1.41 M) for 15.75 h, HCl (7.49% vol) for 2.01 h, and at a deacetylation temperature of 81.15 °C. The resulting chitosan exhibited a degree of deacetylation (DD%) exceeding 93.98%, as confirmed by Fourier-transform infrared (FTIR) spectral analysis, indicating its high purity. The extracted chitosan demonstrated a significant synergistic antibiotic effect against *P. aeruginosa* when combined with ceftazidime, enhancing its bactericidal activity by up to 15-fold. In addition, sub-MIC (minimum inhibitory concentration) concentrations of extracted chitosan (10 and 100 µg/mL) successfully reduced the production of pyocyanin and rhamnolipid, as well as the swimming motility, protease activity and biofilm formation ability in comparison to the control group (*P* < 0.05). Moreover, chitosan treatment downregulated the RhlR and LasR genes in *P. aeruginosa* when compared to the control group (*P* < 0.05). The optimized chitosan extract shows significant potential as a coating agent for surgical equipment, effectively preventing nosocomial infections caused by *P. aeruginosa* pathogens.

## Introduction

*Pseudomonas aeruginosa*, a Gram-negative bacterium, is the predominant organism found in hospital settings (Bassetti et al. 2018). The formation of biofilms, along with other virulence factors, in this pathogen is a quorum sensing (QS) mediated process (Moura-Alves et al. 2019). The LasI-LasR and rhlI-rhlR QS systems govern the virulence of *P. aeruginosa* by regulating the secretion of exotoxins and exoenzymes such as protease, alginate, and extracellular polymeric substances, which contribute to biofilm development (Moura-Alves et al. 2019). The growth of biofilms relies on QS-mediated swarming motility controlled by the rhl system and other QS systems in *P. aeruginosa*, which play a crucial role in infection development. Inhibition of QS systems can attenuate bacterial virulence, as they are key players in pathogenicity (Chadha et al. 2022). Consequently, numerous studies have investigated QS inhibitors and biofilm inhibitors to mitigate biofilm formation and the associated virulence factors (Aleksić et al. 2017; Gökalsın et al. 2017). Given the escalating prevalence of drug-resistant biofilms and their resistance to conventional antibiotics, there is an urgent need for natural compounds that can serve as both QS inhibitors and biofilm inhibitors.

Chitosan, a natural marine polysaccharide macromolecule, is derived from the shells of crustaceans and possesses excellent bioactive properties (Younes and Rinaudo 2015). It is an aminopolysaccharide composed of glucosamine and n-acetyl glucosamine residues obtained through the deacetylation process of chitin found in crab and shrimp shells (Younes and Rinaudo 2015). Due to its biodegradability, biocompatibility, and non-toxicity, chitosan has various applications and exhibits properties such as antimicrobial, immunoadjuvant, antioxidant, antitumor, antithrombogenic, and anticholesteremic effects (Yin et al. 2021). Numerous studies have confirmed its antimicrobial activity against both Gram-positive and Gram-negative bacteria, likely attributed to the interaction between positively charged chitosan molecules and negatively charged bacterial cell membranes (Kamjumphol et al. 2018; Li and Zhuang 2020). Furthermore, chitosan has demonstrated antibiofilm activity against biofilms formed by both Gram-positive and Gram-negative bacteria (Khan et al. 2020; Li and Zhuang 2020). However, the potential of chitosan as a QS inhibitor has not been investigated in the aforementioned studies. Additionally, understanding the efficiency of chitosan production processes is essential for enhancing quality and increasing the global economic value of chitosan.

Given the arduous and time-consuming nature of chitosan extraction methods, as well as their potential for low yields, it is imperative to optimize and establish appropriate process parameters for achieving commercial-scale chitosan production. Additionally, the demand for chitosan in numerous industrial applications has fostered interest in producing it in bulk to facilitate the availability of its raw material, derived from waste generated by the fishery industry. Process design and modeling commonly encounter a multitude of solutions, some of which may be infinite. To identify the best solution within the design region, optimization relies on effective and robust quantitative methods. Essentially, optimization involves selecting the most favorable course of action from a range of available alternatives. Response Surface Methodology (RSM) is one such optimization approach employed to establish the relationship between the dependent response variable and the independent process variables. It elucidates the synergistic and antagonistic effects exerted by these independent variables on the response. Analysis of Variance (ANOVA) further examines the significant and insignificant factors within the experimental range.

This study has two main objectives. Firstly, it aims to optimize and estimate the optimal operating conditions for producing chitosan from the white shrimp species (*Metapenaeus affinis*) while modeling various responses. Secondly, it aims to investigate the anti-QS properties of extracted chitosan against *Pseudomonas aeruginosa*. This evaluation includes assessing its impact on *P. aeruginosa’s* QS-dependent phenotype, as well as the expression of QS-regulated lasR and rhlR genes, and biofilm formation.

## Materials and methods

### Sample collection

Fresh samples of white shrimp (*Metapenaeus affinis*) were procured from three distinct local markets and subsequently packed in plastic bags. The samples were then stored at a temperature of 4 °C until utilized for further analysis. Local individuals proficient in shrimp identification were responsible for verifying the authenticity of the samples.

### Chemical process of chitin extraction

Demineralization is commonly accomplished through the application of acid treatments, which effectively dissolve the calcium carbonate and calcium chloride components present in shrimp shells. Specifically, a 4% concentration of hydrochloric acid is utilized for this purpose. The demineralization process entails subjecting the shrimp shells to a 24 h treatment duration, conducted at a room temperature of 25 °C, with a solid to solvent ratio of 15:1 (ml/g). Notably, the emission of carbon dioxide gas (CO_2_) during demineralization serves as a reliable indicator of the mineral content level within the process (Mohammed et al. 2013). Following the demineralization process, the dried shrimp powder is subjected to a 10% sodium hydroxide (NaOH) solution for a duration of 2 h, maintained at a temperature of 70 °C, with a solvent to solid ratio (v/w) of 15:1 ml/g. This step serves the purpose of eliminating any remaining proteins and other organic materials. Subsequently, the mixture is filtered under vacuum and subsequently washed with tap water for a period of 30 min until the solution attains a neutral pH level (pH= 7). Consequently, the resulting product obtained from this process is chitin (Mohanasrinivasan et al. 2014). To eliminate the pigment present in crustacean shell chitin, reagents such as ethanol, ether, sodium hypochlorite (NaOCl), or hydrogen peroxide (H_2_O_2_) are employed. However, it is crucial to ensure that the chosen bleaching reagent does not impact the physicochemical properties of chitin. Hence, in this study, H_2_O_2_ was selected for this purpose (Kumari and Rath 2014). The deacetylation process is employed to eliminate the acetyl group from chitin. Typically, this is accomplished by treating chitin with a concentrated sodium solution (40–50%). The deacetylation is conducted at 100 °C, with a solvent-to-solid ratio of 10/1 (ml/g), and a reaction time of 12 h. Once deacetylation is complete, chitosan is obtained, followed by washing and rinsing with hot distilled water at 90 °C. Subsequently, the material is filtered and oven-dried at 50 °C for 16 h (Benhabiles et al. 2012). The degree of deacetylation (DD%) of the extracted chitosan can be obtained using Eq. (1). Chitosan is a copolymer consisting of N-acetylglucosamine and D-glucosamine units, and % DD is calculated as the molar fraction of N-acetylglucosamine units in its chain.

1 $$\% {\text{DD = }}\left( {{{{\text{n1}}} \mathord{\left/ {\vphantom {{{\text{n1}}} {\left( {{\text{n1 + n2}}} \right)}}} \right. \kern-\nulldelimiterspace} {\left( {{\text{n1 + n2}}} \right)}}} \right) \times 100.$$

For the determination of % DD, both titration method and Fourier Transform Infrared Spectroscopy (FTIR) approach are employed, using Eqs. (2) and (3) respectively.

2 $$\% {\text{DD = }}{{\left( {{\text{c1V1}} \times {\text{c2V2}}} \right)} \mathord{\left/ {\vphantom {{\left( {{\text{c1V1}} \times {\text{c2V2}}} \right)} {\left( {{\text{M}} \times \left( {100 - {\text{W}}} \right)} \right)}}} \right. \kern-\nulldelimiterspace} {\left( {{\text{M}} \times \left( {100 - {\text{W}}} \right)} \right)}} \times 0.016 \times {{100} \mathord{\left/ {\vphantom {{100} {9.94}}} \right. \kern-\nulldelimiterspace} {9.94}}.$$

3 $$\% {\text{DD }} = {\text{ 1}}00{\text{ }} \times {\text{ }}\left( {{{{\text{1}} - {\text{A1655}}} \mathord{\left/ {\vphantom {{{\text{1}} - {\text{A1655}}} {{\text{A345}}0{\text{ }} \times }}} \right. \kern-\nulldelimiterspace} {{\text{A345}}0{\text{ }} \times }}\,\,{1 \mathord{\left/ {\vphantom {1 {1.33}}} \right. \kern-\nulldelimiterspace} {1.33}}} \right).$$

In Eqs. 2 and 3, n1 and n2 represent the average numbers of D-glucosamine and N-acetylglucosamine units, c1 and c2 represent the concentrations of HCl and NaOH, V1 and V2 represent the volumes of HCl and NaOH added, M represents the mass of the sample, and W represents the moisture content. The factor 0.016 in Eq. (2) corresponds to the amount of amino group in 1 M HCl aqueous solution, and the theoretical NH_2_% in chitosan is 9.94%. The absorbance values A1655 and A3450 at wavelengths 1655 cm^-1^ and 3450 cm^-1^, respectively, are calculated independently using Eq. (4):

4 $${\text{A }} = {\text{ 2 }} \times {\text{ log }}\left( {{\text{T}}\% } \right).$$

where A and T represent the absorbance and transmittance, respectively.

### Characterization of chitosan

#### Crystallinity

Chitosan spectra were acquired using Perkin Elmer FTIR 1600 infrared spectroscopy instrument (Perkin Elmer Spectroscopy, USA) over a frequency range of 4000 to 400 cm ^−1^. To prepare the chitosan sample for analysis, it was mixed with KBr and the resulting mixture was dried and pressed to form a homogeneous sample/KBr disc. The absorption bands at 1655 and 3450 cm ^−1^ were utilized to determine the degree of deacetylation (DD%) (Kumari et al. 2015).

### Statistical analysis using a response surfaces design method (RSM)

A statistical analysis utilizing the response surface design method, referred to as RSM, is DD (%) of chitosan extraction, with the ultimate goal of obtaining a highly pure chitosan product. The selected RSM employs a central composite design (CCD), employing a second-degree mathematical model featuring two-way interactions (Ben Seghir and Benhamza 2017). The experimental design comprises thirty-two factorial points, each with three coded levels (−1, 0, and + 1), and three axial points to establish a central point (refer to Table 1).

**Table 1:** Experiment design levels for various parameters

Control factors	Surface		
	−1	0	1
Concentration of HCl, A (vol%)	1.5	4.5	7.5
Time of demineralization, B (h)	1	10.5	20
Concentration of NaOH, C (M)	0.5	1.5	2.5
Temperature of deacetylation, D (°C)	40	60	80
Time of deacetylation, E (h)	1	2	3

Here, A, B, C, D and E correspond to the independent variables outlined in Table 1. The response surface methodology (RMS) implementation employed the Design Expert 12 statistics software and features quadratic terms, as presented in Table 2.

**Table 2:** Experimental runs and responses of degree of deacetylation (% DD) of chitosan extraction

RUN	Input					Output	
	Concentration of HCl, X_1_ (vol%) (A)	Time of demineralization, X_2_ (h) (B)	Concentration of NaOH, X_3_ (M) (C)	Temperature of deacetylation, X_4_ (°C) (D)	Time of deacetylation, X_5_ (h) (E)	Values of % DD	
1	1.5	1	2.5	40	1	68.01	
2	7.5	1	0.5	80	3	93.98	
3	4.5	10.5	0.5	60	2	63.86	
4	4.5	10.5	1.5	80	2	89.42	
5	4.5	10.5	1.5	60	1	58.72	
6	4.5	10.5	1.5	60	2	69.53	
7	1.5	20	0.5	40	1	58.02	
8	4.5	10.5	1.5	60	3	65.22	
9	4.5	1	1.5	60	2	75.55	
10	4.5	10.5	1.5	60	2	68.73	
11	4.5	10.5	1.5	60	2	68.73	
12	1.5	1	0.5	40	3	86.4	
13	4.5	10.5	1.5	40	2	78.71	
14	7.5	10.5	1.5	60	2	67.23	
15	1.5	20	2.5	80	1	84.44	
16	7.5	1	0.5	40	1	56.12	
17	1.5	20	0.5	80	3	69.05	
18	1.5	1	0.5	80	1	64.89	
19	4.5	10.5	2.5	60	2	67.61	
20	4.5	10.5	1.5	60	2	69.63	
21	7.5	1	2.5	80	1	89.99	
22	1.5	20	2.5	40	3	69.62	
23	7.5	20	0.5	40	3	72.51	
24	7.5	20	0.5	80	1	77.11	
25	7.5	20	2.5	80	3	89.51	
26	4.5	10.5	1.5	60	2	68.63	
27	1.5	1	2.5	80	3	64.52	
28	7.5	20	2.5	40	1	68.08	
29	7.5	1	2.5	40	3	73.17	
30	1.5	10.5	1.5	60	2	60.43	
31	4.5	10.5	1.5	60	2	68.63	
32	4.5	20	1.5	60	2	74.58	

*Pseudomonas aeruginosa* ATCC 10,145 was obtained from the microbiology section of the Institute Pasteur in Tehran, Iran. The microorganism was preserved as a stock culture in a 50% glycerol solution (Pars Azma, Iran) at − 70 °C. Prior to each experiment, the cryopreserved culture was cultured in liquid LB medium (Sigma, UK) at 37 °C and 150 rpm for 24 h until it reached 0.5 OD at 595 nm (1 × 10^7^CFU ml ^− 1^).

### Chitosan synergistic effect with antibiotic

The antimicrobial activity of ceftazidime antibiotics against *P. aeruginosa* was assessed using the macro dilution method. The minimum inhibitory concentrations (MICs) were determined by dissolving the antibiotic in distilled water to create a stock concentration of 10,000 µg/ml. The stock concentration was then filtered through a 0.22 μm Millipore filter. Serial dilutions of the antibiotic were prepared in nutrient broth, ranging from 1 to 5000 µg/ml. Sterile-capped test tubes were inoculated with 100 µl of a bacterial suspension containing 10^4^ cfu/ml. Additionally, 50 µl from each dilution was placed in 7 mm diameter wells on Mueller-Hinton agar medium. Subsequently, all tubes and plates were incubated at 37 °C for 18–24 h. The lowest concentration of antibiotic that showed no visible growth in tubes or inhibition zones in plates was recorded as the MIC. To test the combination of chitosan extract with ceftazidime, the same methods as mentioned above were followed. In this case, 100 µl of bacterial suspension and chitosan were added separately to the serial dilutions of the antibiotic. Once again, 50 µl from each dilution was placed in wells on Mueller-Hinton agar medium, followed by incubation at 37 °C for 18–24 h. The lowest concentration of antibiotic that exhibited no visible growth in tubes or inhibition zones in plates was considered as the MIC.

### Inhibition of virulence factors by Chitosan

#### Pyocyanin extraction and quantification

Pyocyanin extraction in Pseudomonas Broth (PB) was carried out using a previously described method. PB medium consisted of MgCl_2_ (1.4 g/l), peptone (20 g/l), and K_2_SO_4_ (10 g/l) (26). P. aeruginosa was cultivated in PB medium with different concentrations of chitosan extract (10 and 100 µg/mL) for 18 h. The supernatant (5 mL) from the overnight culture was extracted with chloroform (3 mL), and the resulting organic phase was re-extracted with 0.2 N HCl (1.2 mL). The absorbance of the pink aqueous phase was measured at 520 nm. To determine the pyocyanin concentration, the absorbance was multiplied by 17.072, as previously stated (Qais et al. 2019).

### Rhamnolipid assessment

Rhamnolipid production was quantified using the Blue Agar Plate (Bap) method, following a previously established procedure (Bhat et al. 2015). The detection of rhamnolipid was conducted using a mineral salt agar medium supplemented with 2% glucose, 0.05% cetyltrimethylammonium bromide, and 0.02% methylene blue. Each treatment utilized a 0.5 McFarland’s suspension prepared from 24 h bacterial culture, to which sterile chitosan was added in concentrations of 10 and 100 µg/L. Cork borers were used to create 4 mm diameter wells on methylene blue agar plates, which were then loaded with 30 µL of fresh culture from individual isolates. The plates were incubated at 37 °C for 48–72 h. Positive results were observed as a dark blue halo zone around the culture and assessed using a transilluminator at a wavelength of 365 nm.

### Motility assays

The previously described method was employed to evaluate the inhibition of bacterial motility on agar soft plates (Qais et al. 2019). To assess motility, 0.5% LB agar plates were prepared with varying concentrations of chitosan extract (10 and 100 µg/mL). A 5 µL overnight culture was spot-inoculated at the center of each plate, which was subsequently incubated for 18 h. Additionally, swimming motility was evaluated using 0.3% LB agar plates, following the same procedure. The diameter of the swarmed or swam area was measured and reported in millimeters.

### Exoprotease activity

Exoprotease activity was assessed using the azocasein degradation assay, following a previously reported method (Husain et al. 2017). *P. aeruginosa* was cultured with and without chitosan extract supplementation (10 and 100 µg/mL). The cell-free supernatant was obtained by centrifugation. One hundred microliters of the supernatant were mixed with 0.3% azocasein (1000 µl) in a 0.05 M Tris-HCl solution containing 0.5 mM CaCl_2_ at pH 7.5. The reaction mixture was incubated at 37 °C for 15 min, followed by the addition of 500 µl of trichloroacetic acid (10% w/v) to stop the reaction. After centrifugation at 12,000 rpm for 10 min, the optical density of the supernatant was measured at 400 nm.

### Expression of quorum sensing genes

We performed gene assays to evaluate the impact of chitosan on the expression of QS-regulated lasR and rhlR genes. The *P. aeruginosa* isolate was cultivated in LB medium with or without chitosan at a concentration of 100 µg /ml. RNA was isolated using TRIzol reagent (Sigma–Aldrich, UK) and dissolved in 20 µl of 0.1% diethylpyrocarbonate (DEPC)-treated water. On the same day, cDNA was amplified using cDNA Synthesis kit (Yekta Tajhiz Azma Co, Iran) according to the manufacturer’s instructions. The quantitative polymerase chain reaction (qPCR) reactions were conducted using the 7500 Sequence Detection System (Applied Biosystems Inc., Foster, CA, USA) and Power SYBR Green PCR Master Mix (Applied Biosystems). Gene-specific primers were adopted from (Muslim et al. 2018) listed in Table 3 were employed to determine the expression level of QS-regulated genes. The rpsL gene was utilized as a control to normalize the expression of lasR and rhlR genes, and the experiments were carried out in triplicate for real-time analysis.

**Table 3:** Primers and probes employed in this study for real-time analysis

Gene	Primer sequence	Probe sequence	References
rpsL	5′—CTTCCGGGTGTGCGTTAC—3′ 3′—CCCTGCTTACGGTCTTTGAC—5′	CTGGACAC	(Muslim et al. 2018)
rhlR	5′—GTTGCATGATCGAGTTGCTG—3′ 3′—CAGACCGGGTTGGACATC—5´	CCTGGAGC	(Muslim et al. 2018)
lasR	5′—GATATCGGTTATCTGCAACTGCT—3´ 3′—CCGCCGAATATTTCCCATA—5′	GAAGCCAA	(Muslim et al. 2018)

### Biofilm formation

The antimicrobial properties of chitosan were assessed using a microtitre plate assay (26). Bacteria cultured overnight were inoculated into the wells of a 96-well microtitre plate. Chitosan at concentrations of 10 and 100 µg/mL was applied, while control wells remained untreated. Following a 24 h incubation, the media was aspirated, and the wells were rinsed thrice with sterile phosphate buffer. Then, crystal violet solution (0.1%) was added to the wells and allowed to incubate for 15 min. Excess dye was removed by washing the wells, and the biofilms were dissolved in 90% ethanol. The optical density of the dissolved biofilms was measured at 620 nm using a microtitre plate reader.

### Statistical analysis

The experiments were performed in triplicate. The presented data in this study represent the mean values with standard deviation. Statistical analysis was conducted using SPSS software (ver. 24) to compare the control group with the treated groups, employing a one-way ANOVA and independent t-test. A significance level of *P* ≤ 0.05 was used to determine statistical significance.

## Results

### Response surface experiment results

**Fig. 1:**
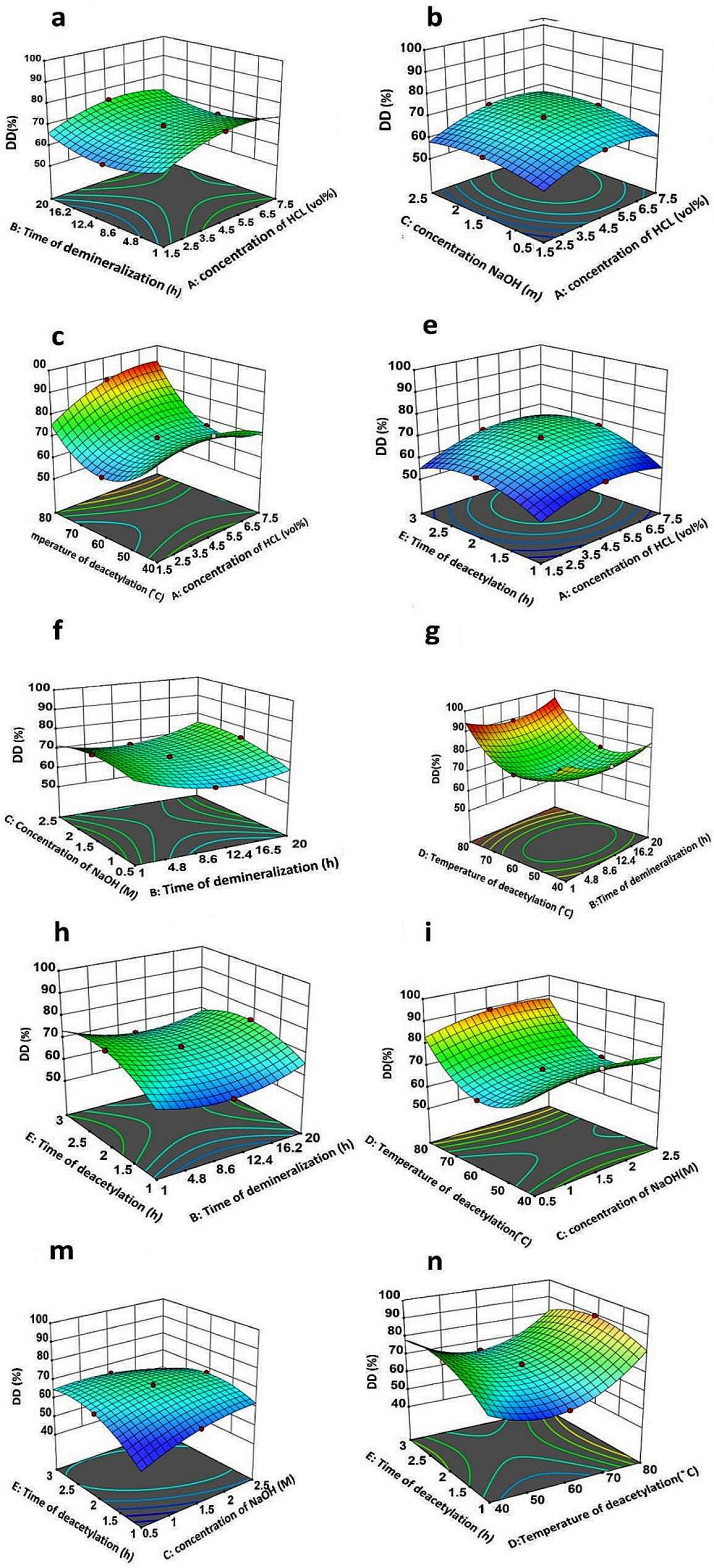
Response surface 3D-plot of optimization of degree of deacetylation (% DD) of chitosan extraction

Figure 1 present the response surface as a 3-D plot, enabling the prediction of DD% for varying test variable values and the identification of their interaction. The interaction between all variables in the DD% response is found to be statistically significant (Table 4).

**Table 4:** The variance analysis of main responses of optimization chitosan extraction from shrimp

Source	Sum of squares	df	Mean square	F-value	*p*-value
Model	2989.35	20	149.47	1250.78	< 0.0001
A-Concentration of HCl	215.77	1	215.77	1805.59	< 0.0001
B-Time of demineralization	5.24	1	5.24	43.83	< 0.0001
C-Concentration of NaOH	60.54	1	60.54	506.59	< 0.0001
D-Temperature of deacetylation	472.99	1	472.99	3958.08	< 0.0001
E-Time of deacetylation	190.78	1	190.78	1596.46	< 0.0001
AB	0.7056	1	0.7056	5.90	0.0334
AC	10.24	1	10.24	85.69	< 0.0001
AD	398.60	1	398.60	3335.60	< 0.0001
AE	34.93	1	34.93	292.29	< 0.0001
BC	103.33	1	103.33	864.67	< 0.0001
BD	30.80	1	30.80	257.76	< 0.0001
BE	42.32	1	42.32	354.10	< 0.0001
CD	19.36	1	19.36	162.01	< 0.0001
CE	395.02	1	395.02	3305.60	< 0.0001
DE	161.54	1	161.54	1351.85	< 0.0001
A²	69.03	1	69.03	577.66	< 0.0001
B²	86.78	1	86.78	726.23	< 0.0001
C²	28.30	1	28.30	236.85	< 0.0001
D²	549.15	1	549.15	4595.45	< 0.0001
E²	126.03	1	126.03	1054.63	< 0.0001
Residual	1.31	11	0.1195		
Lack of fit	0.2195	6	0.0366	0.1670	0.9750
Pure error	1.10	5	0.2190		
Cor Total	2990.66	31			

**Fig. 2:**
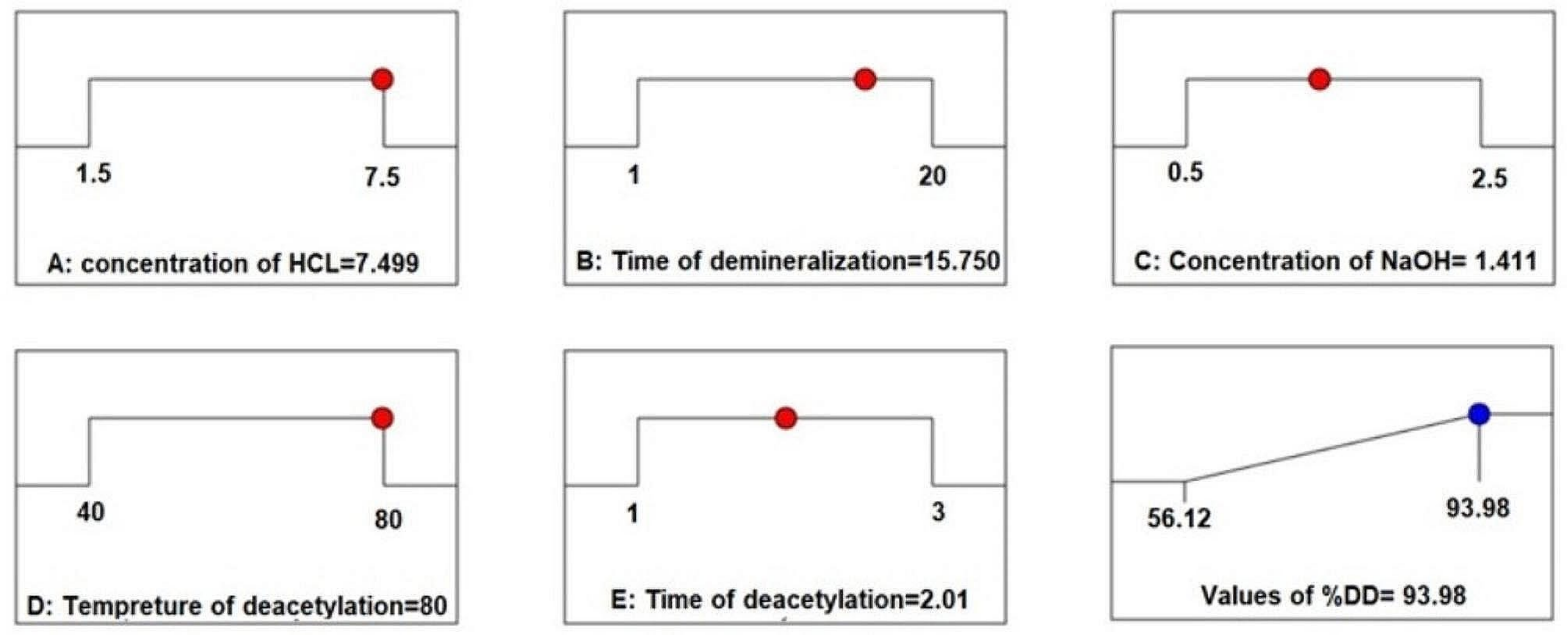
Desirability ramp for optimization of (% DD) of chitosan extraction

In order to optimize and validate the predicted mathematical model, additional Expert Design runs were conducted using the same experimental conditions. The accuracy of the model was significantly enhanced by increasing the desirability function. The optimal parameters, corresponding to a desirability value of 0.99, resulted in a DD% of 93.98% (Fig. 2). Under the specified conditions, the highest achieved DD% was attained with the following parameters: NaOH concentration (1.41 M), demineralization time (15.75 h), HCL concentration (7.49% vol), deacetylation time (2.01 h), and deacetylation temperature (81.15 °C). Chitosan samples were prepared using these conditions and subjected to characterization analysis.

### FTIR characterization of extracted chitosan

**Fig. 3:**
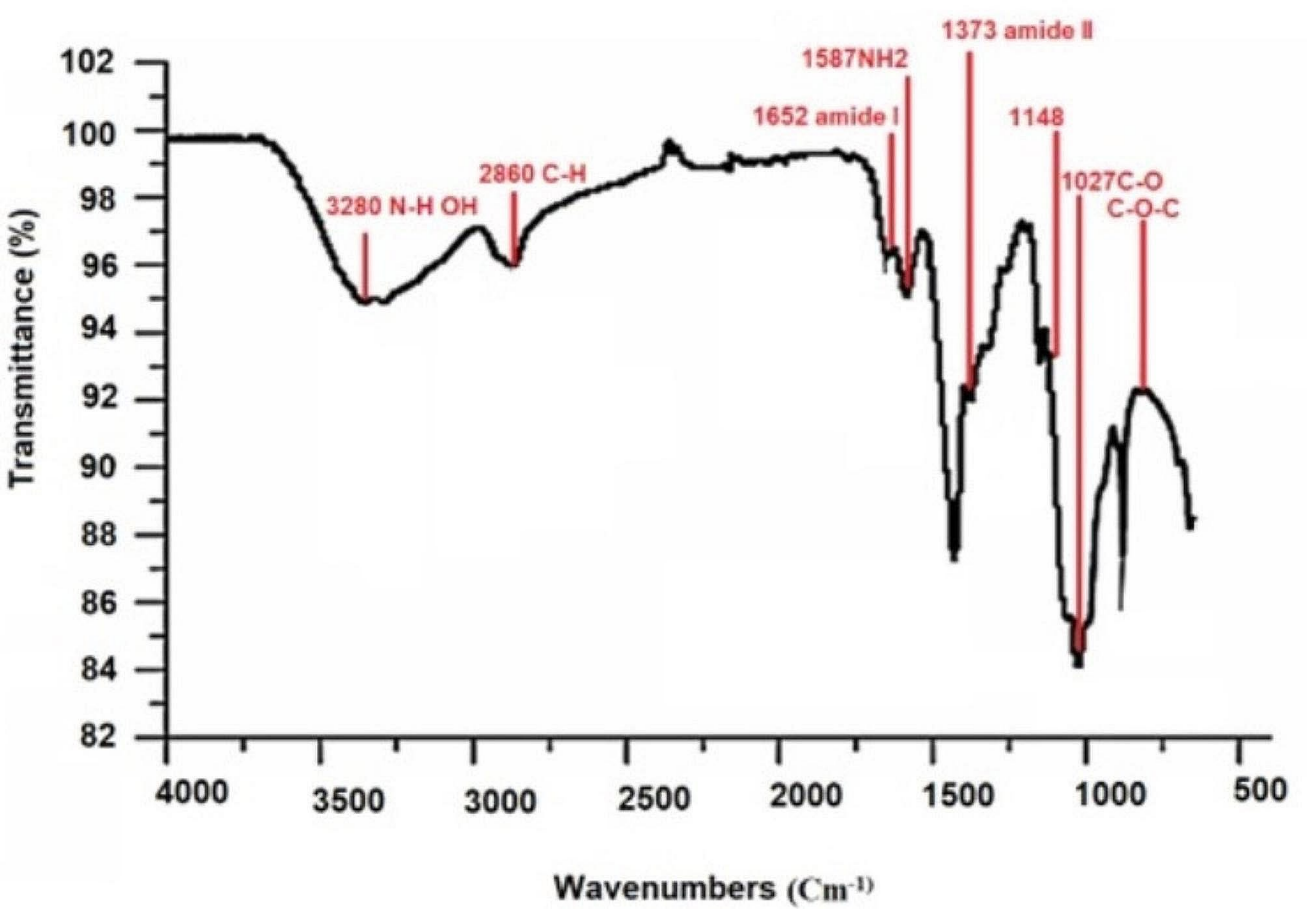
Fourier transform infrared spectroscopy (FTIR) of extracted chitosan from shrimp

Chitosan spectra were obtained using FTIR. The main peaks are depicted in Fig. 3. Analysis of the spectrum reveals a broad band between 3100 and 3500 cm^−1^, which corresponds to stretching vibrations of water, hydroxyl groups, and free amino groups (–OH and –NH).

### Antibiotic activity of chitosan

The antibacterial activity of ceftazidime against *P.aeruginosa* was found to be weak with MICs of 1021 µg/ml. However, when combined with chitosan, ceftazidime exhibited strong antibacterial activity. The MICs of ceftazidime decreased to 65 µg/ml. Similar results were obtained using the agar diffusion method, with inhibition zones reaching 25 mm at 64 µg/ml (Table 5).

**Table 5:** Minimum inhibitory concentrations (MICs) of ceftazidime against *Pseudomonas aeruginosa* determined in both tube and plate assays, with and without the addition of extracted chitosan

Antibiotic	MIC (µg/ml)	Diameter of inhibition zone (mm)
Ceftazidime	1021	19
Samacycline + chitosan	65	25

### Effect of chitosan on quorum sensing

**Fig. 4:**
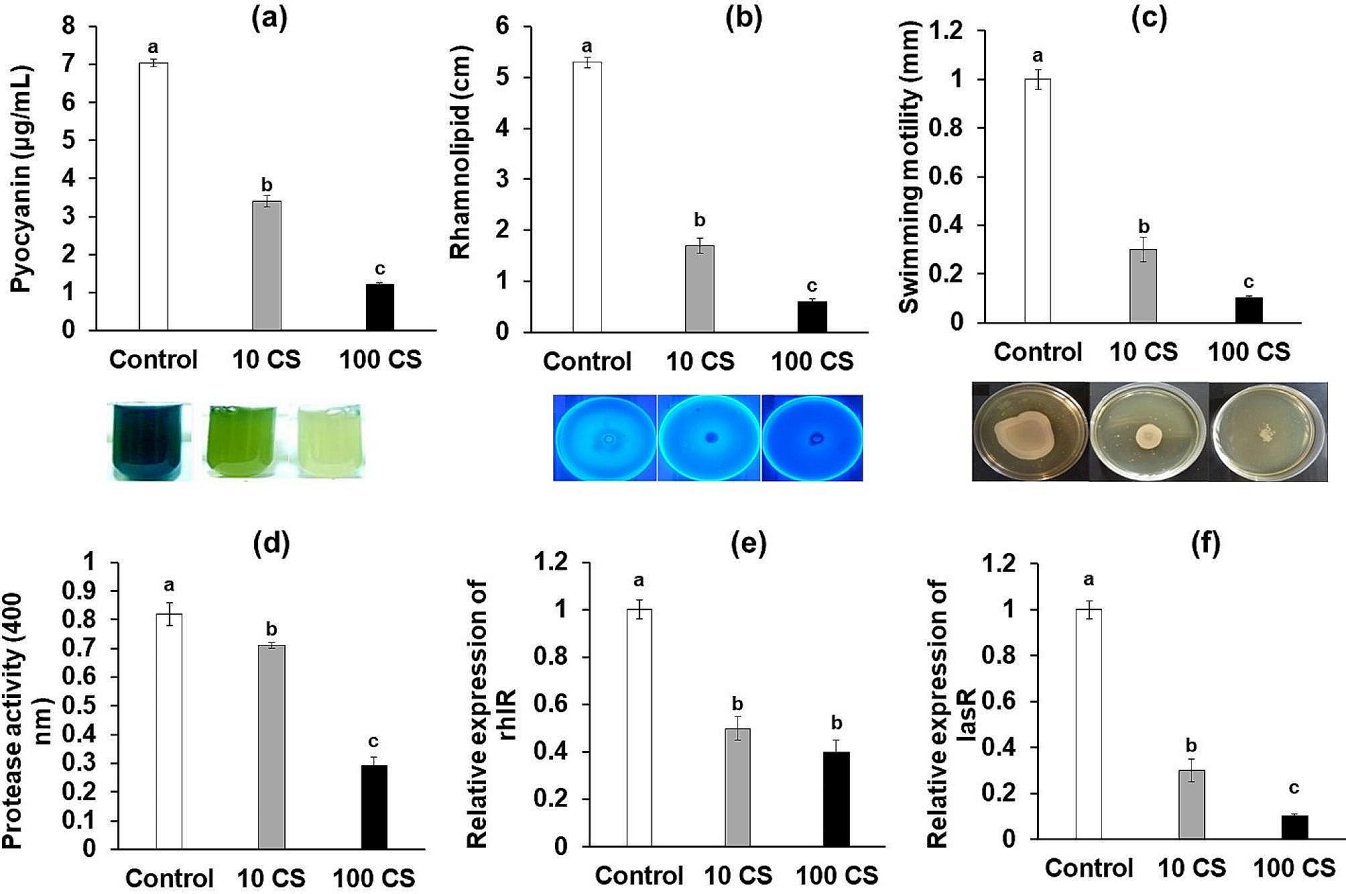
The impact of sub-minimum inhibitory concentration (MIC) concentrations of shrimp-derived chitosan (CS) on the factors involved in quorum sensing of *Pseudomonas aeruginosa*. (**a**): pyocyanin production, (**b**): rhamnolipid production, (**c**): swimming motility, (**d**): protease activity, (**e** and **f**): expression of rthIR and lasR genes. Significant differences among the groups are denoted by distinct letters (*P* < 0.05) (*n* = 6)

The results demonstrated that treatment of *Pseudomonas aeruginosa* bacteria with chitosan concentrations of 10 and 100 µg/mL led to a significant decrease in the production of pyocyanin and rhamnolipid, as well as a reduction in swimming motility and protease activity compared to the control group (*P* < 0.05) (Fig. 4). The greatest decrease was observed at the concentration of 100 µg/mL chitosan. Furthermore, treatment with chitosan resulted in a downregulation of the RhlR and LasR genes in P. aeruginosa when compared to the control group (*P* < 0.05) (Fig. 4).

#### Effect of chitosan on biofilm formation

**Fig. 5:**
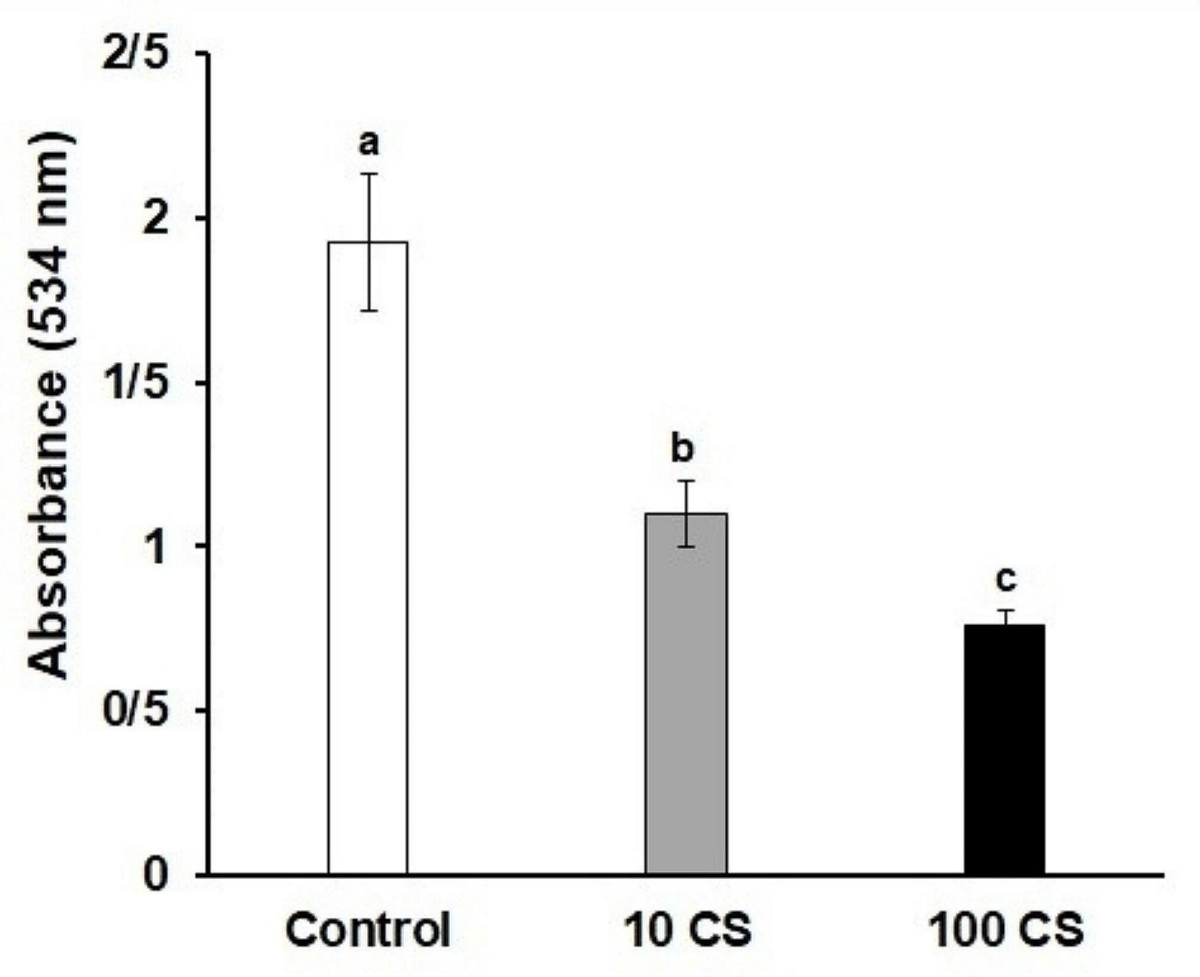
The impact of sub-MIC concentrations of CS on biofilm formation of *Pseudomonas aeruginosa*

Chitosan treatment significantly impaired the biofilm-forming capacity of *Pseudomonas aeruginosa*, particularly when administered at a dose of 100 µg/mL (Fig. 5). This resulted in a noteworthy reduction in biofilm formation capability (*P* < 0.5) (Fig. 5).

## Discussion

In this study, we optimized the extraction of chitosan from the white shrimp species (*Metapenaeus affinis*) by manipulating concentration, temperature, reaction time, and v/w ratio. Our results showed an impressive DD% of 93.98%, surpassing previous studies conducted with different material sources. Unlike most previous research that focused on only three variables (Hwang et al. 2002; Younes et al. 2014), our study considered five significant variables for optimization. Based on the results of Table 2 the best concentration of HCL and NaOH are 7.5 (vol %) and 1 M was obtained in the demineralization time of 1 h and the distillation time of 3 h with the distillation temperature 80 ( °C). The results of FTIR analysis confirmed the authenticity of the extracted chitosan. The peak at 2860 cm^−1^ indicates stretching vibrations related to the branching of C–H bonds, specifically attributed to CH2 and CH3 groups (Kumirska et al. 2010). The 1652 cm^−1^band corresponds to the amide I absorption resulting from interactions between hydrogen and hydroxyl groups, indicating the removal of the acetyl group. At 1587 cm^−1^, the band corresponds to amide II (–NH2 bending). The peak observed at 1429 cm^−1^ represents C–H stretching, while the band at 1373 cm^−1^ corresponds to amide III and signifies C-N stretching of N-acetyl-glucosamine. The absorption peak at 1148 cm^−1^ indicates the presence of a symmetric glycosidic linkage (C–O–C), and at 1027 cm^−1^, an absorption band is observed, indicating stretching vibrations of the C–O ring. The absorption peak around 880 cm^−1^ is attributed to the β-anomer (1–4) glycosidic linkage (C–O–C) (Abdel-Rahman et al. 2015).

The present study demonstrated the synergistic effect of combined chitosan and antibiotic treatment, resulting in a decreased minimum inhibitory concentration (MIC) in *P. aeruginosa*. This finding highlights the antimicrobial efficacy of chitosan. Ceftazidime, a member of the β-Lactam antibiotics group, is widely recognized as one of the most effective and safe antibiotics. This group encompasses numerous antibiotics that share the common β-Lactam ring structure and can be further classified into five main subgroups based on various criteria (Brooks et al. 2014). These bactericidal antibiotics exert their mode of action by inhibiting cell wall synthesis, specifically by interfering with the transpeptidation reaction through covalent binding to the target site of Penicillin-binding proteins (PBPs). This binding ultimately halts the synthesis of peptidoglycan, leading to bacterial cell death (Guilfoile and Alcamo 2007). It is important to note that β-Lactam antibiotics are effective against actively growing bacteria that are in the process of synthesizing their cell walls (Drlica and Fong 2008). The antimicrobial properties of chitin, chitosan, and their derivatives have gained significant attention in recent years, particularly in their effectiveness against various microorganisms. Several mechanisms have been proposed to explain the inhibitory effects of chitosan on microbial cells. These multiple mechanisms contribute to the antimicrobial efficacy of chitosan and its potential as an effective agent in combating microbial infections. chitosan’s polycationic nature enables it to interact with anionic groups on the cell surface. This interaction forms an impermeable layer surrounding the cell, which hinders the transport of essential solutes through the outer membrane of gram-negative bacteria. Consequently, it induces structural changes in the cell membrane, increases permeability, and causes the leakage of proteins and other intracellular components (Kamala et al. 2013). chitosan acts as a chelating agent, selectively binding trace metals. This subsequently inhibits toxin production and microbial growth (Muslim et al. 2018). chitosan triggers various defense processes in the host tissue, acts as a water-binding agent, and inhibits specific enzymes. Low molecular weight (LMW) chitosan is capable of entering the cytosol of microorganisms (Kašparová et al. 2022). Upon binding with DNA, it interferes with mRNA and protein synthesis. chitosan forms an impermeable polymeric layer on the cell surface, altering cell permeability and preventing nutrient uptake. Furthermore, chitosan has the ability to adsorb electronegative substances within the cell, leading to their flocculation. This disturbance of the microorganism’s physiological activities ultimately results in cell death (Badawy and Rabea 2011). chitosan possesses a broad spectrum of antimicrobial properties, but its practical application is hindered by its low solubility at neutral pH. Nonetheless, research has demonstrated that enhancing the positive charge of chitosan enables stronger binding to bacterial cell walls (Kamala et al. 2013). The antimicrobial efficacy of chitosan correlates directly with its degree of deacetylation, which determines the number of amino groups present (Kim et al. 2003). Consequently, a higher degree of deacetylation leads to greater quantities of protonated amino groups in acidic conditions. This increased solubility allows for enhanced interaction between chitosan and negatively charged cell walls of microorganisms, thereby augmenting its antimicrobial activity (Silva et al. 2021).

QS genes, which frequently encode virulence factors, play a crucial role in the interaction between bacteria and their hosts (Warrier et al. 2021). The formation of QS-mediated biofilms confers antibiotic resistance to bacteria and impedes the effectiveness of the host immune (Warrier et al. 2021). In *P.aeruginosa*, the lasR and rhlR systems govern QS and are responsible for the synthesis of various virulence enzymes, including LasA protease, elastase, pyocyanin, and rhamnolipids (Qin et al. 2022). In this study, chitosan extract effectively reduced the production of pyocyanin, rhamnolipids, and protease in *P. aeruginosa*. It also decreased the expression of lasR and rhlR, as well as swimming motility and biofilm formation in a dose-dependent manner. Similar findings were observed by Rubini et al. (2019)., where pyocyanin production in clinical isolates of *P. aeruginosa* was reduced by 40–80% upon treatment with chitosan extract from crab shell (*Pseudomonas sanguinolentus*) (Rubini et al. 2019). This confirms chitosan’s capability to disrupt the rhl quorum sensing system, which regulates pyocyanin pigment production (Le Berre et al. 2008). Additionally, a previous report by Overhage et al. (2007) demonstrated that a swarming-associated gene in *P. aeruginosa* is responsible for biofilm formation and the production of virulence enzymes (Overhage et al. 2007). The results clearly demonstrate that extracted chitosan has a significant inhibitory effect on the swarming motility of *P. aeruginosa* strains, leading to the prevention of biofilm formation.

In this investigation, the extracted chitosan demonstrated a significant decrease in the ability of *P. aeruginosa* to form biofilms at sub-MIC levels. The presence of cationic charge in chitosan facilitates its penetration into the biofilm, disrupting the ionic charge of the cell membrane and preventing adherence to both living and non-living surfaces (Zhang et al. 2013). A study by Orgaz et al. (2011) showed that mature biofilms of Pseudomonas spp. are highly susceptible to chitosan treatment. Consistent with this finding, extracted chitosan exhibited a powerful effect against preformed biofilms of *P. aeruginosa*, reinforcing its potential as an antibiofilm agent (Orgaz et al. 2011). The biofilm matrix consists of various macromolecules, with the extracellular polysaccharide substance (EPS) playing a crucial role in the three-dimensional structure of biofilms (Limoli et al. 2015). EPS has been implicated in limiting the entry of antibiotics into the biofilm matrix, contributing to antibiotic resistance (Pinto et al. 2020). Extracted chitosan disrupts the structural integrity of the biofilm architecture by reducing the EPS layer (Rubini et al. 2019). Mu et al. (2014) reported the antibiofilm efficacy of chitosan, which resulted in a reduction in biomass of Listeria species through biofilm dispersal (Mu et al. 2014).

In conclusion, the current study demonstrates the successful extraction of highly deacetylated chitosan from white shrimp (*Metapenaeus affinis*) shells. Additionally, the research highlights the utility of RSM as a valuable statistical approach for investigating the impact of various independent parameters on chitosan preparation. These parameters include HCl concentration, demineralization time, NaOH concentration, deacetylation temperature, and deacetylation time. The highest degree of deacetylation achieved in this study was 93.98%, and the purity of the chitosan was confirmed using FTIR analysis. The extracted chitosan exhibited remarkable antibacterial properties, along with a synergistic effect when combined with ceftazidime. It also demonstrated anti-quorum sensing and anti-biofilm activities against *P. aeruginosa*. Therefore, the optimized chitosan extract holds great potential as a coating agent for surgical equipment and indwelling catheters, effectively preventing nosocomial infections caused by *P. aeruginosa* pathogens. Additionally, it can find applications in the food packaging industry and cosmetics sector.

## Data Availability

Data are available upon reasonable request.

## Abbreviations

ANOVA: Analysis of variance
Bap: Blue agar plate
CCD: Central composite design
CO2: Carbon dioxide gas
DD: Degree of deacetylation
DEPC: Diethylpyrocarbonate
EPS: Extracellular polysaccharide substance
FTIR: Fourier transform infrared spectroscopy
H2O2: Hydrogen peroxide
LMW: Low molecular weight
MICs: Minimum inhibitory concentrations
NaOH: Sodium hydroxide
NaOCl: Sodium hypochlorite Penicillin-binding proteins
PBPs: Penicillin-binding proteins
PB: Pseudomonas broth
qPCR: Quantitative polymerase chain reaction
QS: Quorum sensing
RSM: Response surface methodology
