# Correction: Optimizing chitosan derived from *Metapenaeus affinis*: a novel anti-biofilm agent against *Pseudomonas aeruginosa*

**DOI:** 10.1186/s13568-024-01815-z

**Published:** 2025-01-29

**Authors:** Anali Riahi, Hadideh Mabudi, Elahe Tajbakhsh, Laleh Roomiani, Hasan Momtaz

**Affiliations:** 1 Department of Microbiology, Faculty of Basic Sciences, Shahrekord Branch, Islamic Azad University, Shahrekord, Iran; 2 Department of Fisheries, Ahvaz Branch, Islamic Azad University, Ahvaz, Iran

**Correction to: AMB Express (2024) 14:77**

10.1186/s13568-024-01732-1

The original publication of this article (Riahi et al. 2024) contained errors in the affiliations 1 and 2. The incorrect and correct affiliations are listed in this correction article. The original article has been updated.

Incorrect:

^1^Department of Microbiology, Faculty of Basic Sciences, Islamic Azad University, Shahrekord Branch, Shahrekord, Iran

^2^Department of Fisheries, Islamic Azad University, Golestan highway, Farhang Shahr, Ahvaz Branch, PO Box 1915, 61349-37333 Ahvaz, Iran

Correct:

^1^Department of Microbiology, Faculty of Basic Sciences, Shahrekord Branch, Islamic Azad University, Shahrekord, Iran

^2^Department of Fisheries, Ahvaz Branch, Islamic Azad University, Ahvaz, Iran
